# Dietary omega-3 polyunsaturated fatty acids reduce cytochrome c oxidase in brain white matter and sensorimotor regions while increasing functional interactions between neural systems related to escape behavior in postpartum rats

**DOI:** 10.3389/fnsys.2024.1423966

**Published:** 2024-10-31

**Authors:** Carley Rivers, Christopher Farber, Melissa Heath, Elisa Gonzales, Douglas W. Barrett, F. Gonzalez-Lima, Michelle A. Lane

**Affiliations:** ^1^Nutrition and Foods Program, School of Family and Consumer Sciences, Texas State University, San Marcos, TX, United States; ^2^Department of Psychology, The University of Texas at Austin, Austin, TX, United States

**Keywords:** n-3 polyunsaturated fatty acids, pregnancy, white matter, cytochrome c oxidase, somatosensory

## Abstract

**Introduction:**

Previously, we showed that omega-3 polyunsaturated fatty acid n-3 (PUFA) supplementation improved the performance of postpartum rats in the shuttle box escape test (SBET).

**Methods:**

The brains of these rats were used in the current study which examined brain cytochrome c oxidase (CCO) activity in white matter bundles and 39 regions spanning sensorimotor, limbic, and cognitive areas to determine the effects of n-3 PUFAs on neural metabolic capacity and network interactions.

**Results:**

We found that n-3 PUFA supplementation decreased CCO activity in white matter bundles, deep and superficial areas within the inferior colliculus, the anterior and barrel field regions of the primary somatic sensorimotor cortex, the secondary somatic sensorimotor cortex, the lateral, anterior regions of the secondary visual cortex and the ventral posterior nucleus of the thalamus, and the medial nucleus of the amygdala. Structural equation modeling revealed that animals consuming diets without n-3 PUFAs exhibited fewer inter-regional interactions when compared to those fed diets with n-3 PUFAs. Without n-3 PUFAs, inter-regional interactions were observed between the posterior cingulate cortex and amygdala as well as among amygdala subregions. With n-3 PUFAs, more inter-regional interactions were observed, particularly between regions associated with fear memory processing and escape. Correlations between regional CCO activity and SBET behavior were observed in rats lacking dietary n-3 PUFAs but not in those supplemented with these nutrients.

**Discussion:**

In conclusion, consumption of n-3 PUFAs results in reduced CCO activity in white matter bundles and sensorimotor regions, reflecting more efficient neurotransmission, and an increase in inter-regional interactions, facilitating escape from footshock.

## 1 Introduction

Many foods in the Western diet are low in omega-3 polyunsaturated fatty acids (n-3 PUFAs), resulting in decreased intake of these essential lipids (Simopoulos, 1999, 2002; Baker et al., 2016). Found at high levels in the brain (Rapoport et al., 2007; Betsholtz, 2015), these essential fatty acids are depleted in mothers during gestation and lactation when they are used to support fetal and infant brain development (Billeaud et al., 2021). To remediate this deficiency, supplemental n-3 PUFAs are recommended for pregnant and lactating women. While research regarding the impact of n-3 PUFAs on the brain often explores the neurodevelopmental effects of n-3 PUFAs beginning at conception, these nutrients also impact the fatty acid composition and function of the adult brain, the focus of the current study. For example, using a postpartum model similar to ours, Levant et al. found that rat dams fed a low alpha-linolenic acid (ALA) diet for two cycles of gestation and lactation experienced a depletion of brain DHA, particularly in the frontal cortex and temporal lobe, as compared to female, virgin rats fed the same diet (Levant et al., 2006, 2007). Decreased DHA was also associated with decreased hippocampal brain-derived neurotrophic factor (BDNF) gene expression (Levant et al., 2008). Similarly, lack of dietary ALA interacted with reproductive status to reduce cortical DHA and alter the number of dopamine D_2_-like receptors in the ventral striatum (including the nucleus accumbens and olfactory tubercle), but not the caudate-putamen of virgin or biparous rats (Davis et al., 2010). More recent work supports these findings, showing that PUFA ratios in the hippocampus, prefrontal cortex, and hypothalamus mirror those in the diet of adult, male, rats (Horman et al., 2020).

In addition to altering regional brain lipid composition, dietary PUFAs exert anti-inflammatory and immunomodulatory effects that positively impact the adult brain (Kusy et al., 2024). For example, dietary n-3 PUFAs reduce markers of hypothalamic and hippocampal neuroinflammation in male mice consuming a high fat diet (Sanchez et al., 2024), the hippocampus and amygdala of aged, male rats consuming a processed foods diet (Butler et al., 2021), and in the hippocampus of aged, female mice (Taoro-González et al., 2022). Of relevance, adult females, the target population in the current study, are more sensitive than males to the ability of dietary n-3 PUFA deficiency to decrease the number of microglia and, consequently, hippocampal neurogenesis (Rodríguez-Iglesias et al., 2022). Hippocampal neurogenesis affects a spectrum of behaviors, in particular those related to depression and memory (Toda et al., 2019).

With respect to behavior, in a previous study our group found that multiparous, postpartum rats receiving dietary n-3 PUFA supplementation did not differ from deficient animals in depression-related behaviors, but did exhibit improved adaptive coping as evidenced by an increase in the number of escapes and reduced escape times in the shuttle box escape test (SBET), when compared to rats deficient in n-3 PUFAs (Gonzales et al., 2015). In both humans and rodents, adaptive behavioral coping in response to stressful stimuli protects against depressive disorders (Schneiderman et al., 2005; Patel et al., 2019).

Adaptive behavioral coping depends on a functional fear memory processing system (Heimer and Van Hoesen, 2006). Although there is minimal research on the effect of n-3 PUFAs on specific regions of the fear memory processing system, adequate n-3 PUFA intake, beginning with the mother’s diet during gestation and continuing throughout the lifespan, supports myelination. Myelination is required for appropriate brain function (Prado and Dewey, 2014). For example, n-3 PUFAs appear to protect against disorders associated with disrupted myelination such as psychosis (Collins et al., 2024), schizophrenia (Pawełczyk et al., 2017) multiple sclerosis (Valenzuela et al., 2011; Chen et al., 2014), and traumatic brain injury (Pu et al., 2013; Liu et al., 2016) because they reduce damage to white matter and stimulate the production of myelin proteins (Salvati et al., 2008). We hypothesize that this protection of white matter might consequently improve inter-regional interactions and, hence, the ability of individual brain regions to work together as systems, e.g., the fear memory processing system, affecting behavior.

The objective of the current study was to systematically evaluate how alterations in n-3 PUFA intake in a multiparous, postpartum adult rat model affect cytochrome c oxidase (CCO) activity in white matter bundles and 39 brain regions supporting a variety of sensorimotor, cognitive, and emotional functions. To date, much work concerning how n-3 PUFAs affect the brain has focused on a limited number of regions per study. This piece-meal approach reduces the ability to detect the more wide-spread effects of these nutrients on brain systems involved in a variety of functions. In the current study, the use of CCO histochemistry allowed us to determine how n-3 PUFA intake simultaneously altered regional brain metabolism in numerous regions located throughout the brain, and, importantly, the interactions between these regions, affecting systems involved in behavior. CCO is the last enzyme in the electron transport chain, ultimately producing energy in the form of adenosine triphosphate (ATP) (Padilla et al., 2011). CCO levels can therefore be used to demonstrate the consequence of a chronic manipulation like nutritional supplementation, as CCO activity reflects the metabolic capacity of a given region. Because these brain regions function in connection with each other as networks and not in isolation, CCO staining is an ideal way to elucidate the effect of n-3 PUFAs on inter-regional interactions. CCO histochemistry has been used to map affected brain regions in animal models of various mental health conditions, including depression and attention deficit hyperactivity disorder (Shumake and Gonzalez-Lima, 2003; O'Reilly et al., 2009; Spivey et al., 2009; Padilla et al., 2011). For example, our group has used CCO histochemistry to determine how 13-*cis*-retinoic acid administration alters the inter-regional interactions between brain regions associated with depression-related behavior in adolescent mice (O'Reilly et al., 2009). Similarly, in the current study regional mean differences in CCO activity and structural equation modeling (SEM) were used to identify regions where n-3 PUFAs had the greatest effect. Finally, the use of the brains from animals in our behavioral study allowed for correlation of CCO activity with behavior.

## 2 Materials and methods

### 2.1 Animals

Brains were obtained from the multiparous model described in our previous publication (Gonzales et al., 2015). This model is similar to that developed by Levant et al. (2006, 2007, 2008). Long Evans females were fed either an n-3 PUFA deficient or a n-3 PUFA supplemented diet through two cycles of gestation and lactation, separated by 10 days. The diets consumed and behavioral tests experienced by these rats are detailed in Gonzales et al. (2015). All diets were based on AIN-93G to meet the nutrient requirements of pregnancy and lactation. Diets were manufactured by Research Diets, Inc. (New Brunswick, NJ) to contain either 7% sunflower oil, devoid of n-3 PUFA (without n-3 PUFAs), or 7% menhaden oil containing 14.2% EPA and 10.3% DHA (with n-3 PUFAs). Both diets were identical except for fat source. The standard, facility diet consumed during mating contained 0.2% ALA and negligible amounts of EPA and DHA. Animals were sacrificed via decapitation. Although 10 animals were assigned to each diet, one animal was removed from the final data set due to the presence of multiple outliers discovered during statistical analyses, resulting in n = 10 in the deficient group and n = 9 in the supplemented group. All procedures involving animals were approved by the Institutional Animal Care and Use Committees of the University of Texas at Austin and Texas State University.

### 2.2 Tissue processing

CCO histochemistry was performed as described previously (Shumake et al., 2000; O'Reilly et al., 2009). Briefly, upon sacrifice dam brains were frozen rapidly in isopentane and stored at −40°C. Forty μm cryostat sections were thaw-mounted onto slides and kept frozen at −40°C until processed for CCO histochemistry within a year of sacrifice. To perform CCO histochemical staining, slides were lightly fixed in phosphate, sucrose, glutaraldehyde buffer prior to metal intensification in cobalt chloride solution. CCO staining was assessed after an hour-long incubation in 0.1 M phosphate buffer containing diaminobenzidine tetrahydrochloride, cytochrome c, sucrose, catalase, and dimethyl sulfoxide. Following staining, tissue was fixed in formalin to stop this reaction. The tissues were dehydrated in a series of ethanol baths then cleared with xylene before adhering coverslips using Permount (Fisher Chemical, Waltham, MA).

**Figure 1:**
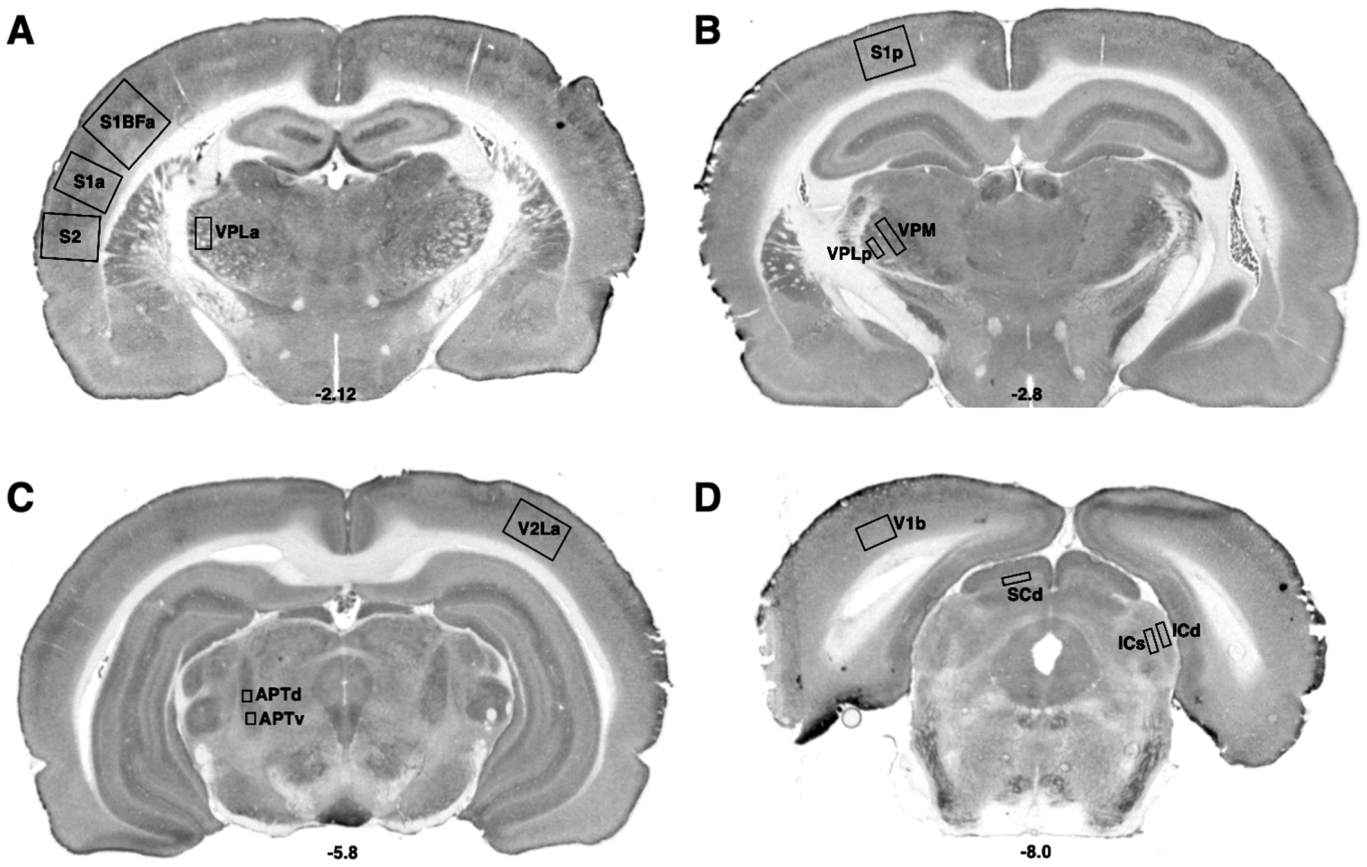
Bregma levels for CCO readings of sensorimotor regions. The Bregma level for each image is shown at the bottom center of that image. Representative images for each Bregma level are shown. **(A)** Ventral posterior nucleus, lateral, anterior measurement (VPLa); primary somatic sensorimotor cortex, barrel field, anterior measurement (S1BFa); primary somatic sensorimotor cortex, anterior measurement (S1a), secondary somatic sensorimotor cortex (S2); **(B)** Ventral posterior nucleus, medial (VPM); ventral posterior nucleus, lateral, posterior measurement (VPLp); primary somatic sensorimotor cortex, posterior measurement (S1p). **(C)** Secondary visual cortex, lateral, anterior measurement (V2La); anterior pretectal nucleus, dorsal (APTd); anterior pretectal nucleus, ventral (APTv). **(D)** Primary visual cortex, binocular (V1b); superior colliculus, deep (SCd); inferior colliculus, superficial (ICs); inferior colliculus, deep (ICd).

**Figure 2:**
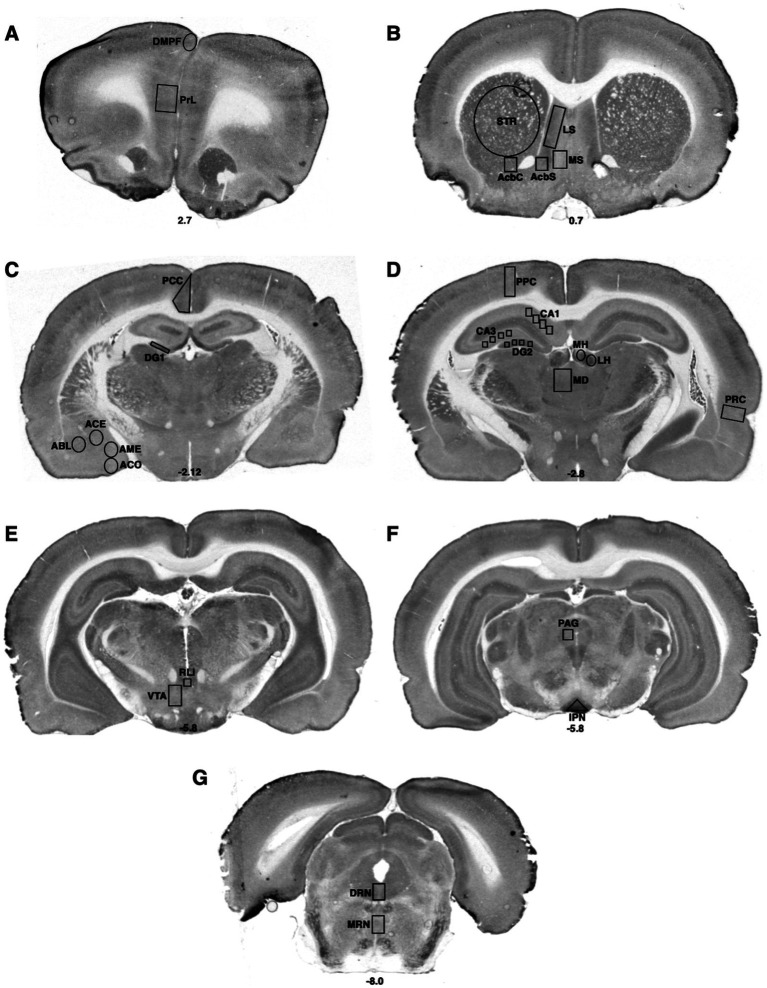
Bregma levels for CCO readings of cognitive and emotional regions. The Bregma level for each image is shown at the bottom center of that image. Representative images for each Bregma level are shown. **(A)** Prelimbic cortex (PrL); dorsal medial prefrontal cortex (DMPF). **(B)** Dorsal striatum (STR); lateral septal nucleus (LS); the medial septal nucleus (MS); shell (AcbS) and core (AcbC) regions of the nucleus accumbens. **(C)** Posterior cingulate cortex (PCC); dentate gyrus (DG); cortical nucleus of the amygdala (ACO); central nucleus of the amygdala (ACE); basolateral nucleus of the amygdala (ABL); medial nucleus of the amygdala (AME). **(D)** Posterior parietal cortex (PPC); hippocampal CA1 region (CA1); hippocampal CA3 region (CA3); dentate gyrus (DG); medial habenula (MH); lateral habenula (LH); mediodorsal nucleus of the thalamus (MD); perirhinal cortex (PRC). **(E)** Rostral linear raphe nucleus (RLi); ventral tegmental area (VTA). **(F)** Periaqueductal gray (PAG); interpeduncular nucleus (IPN). **(G)** Dorsal raphe nucleus (DRN); medial raphe nucleus (MRN).

**Figure 3:**
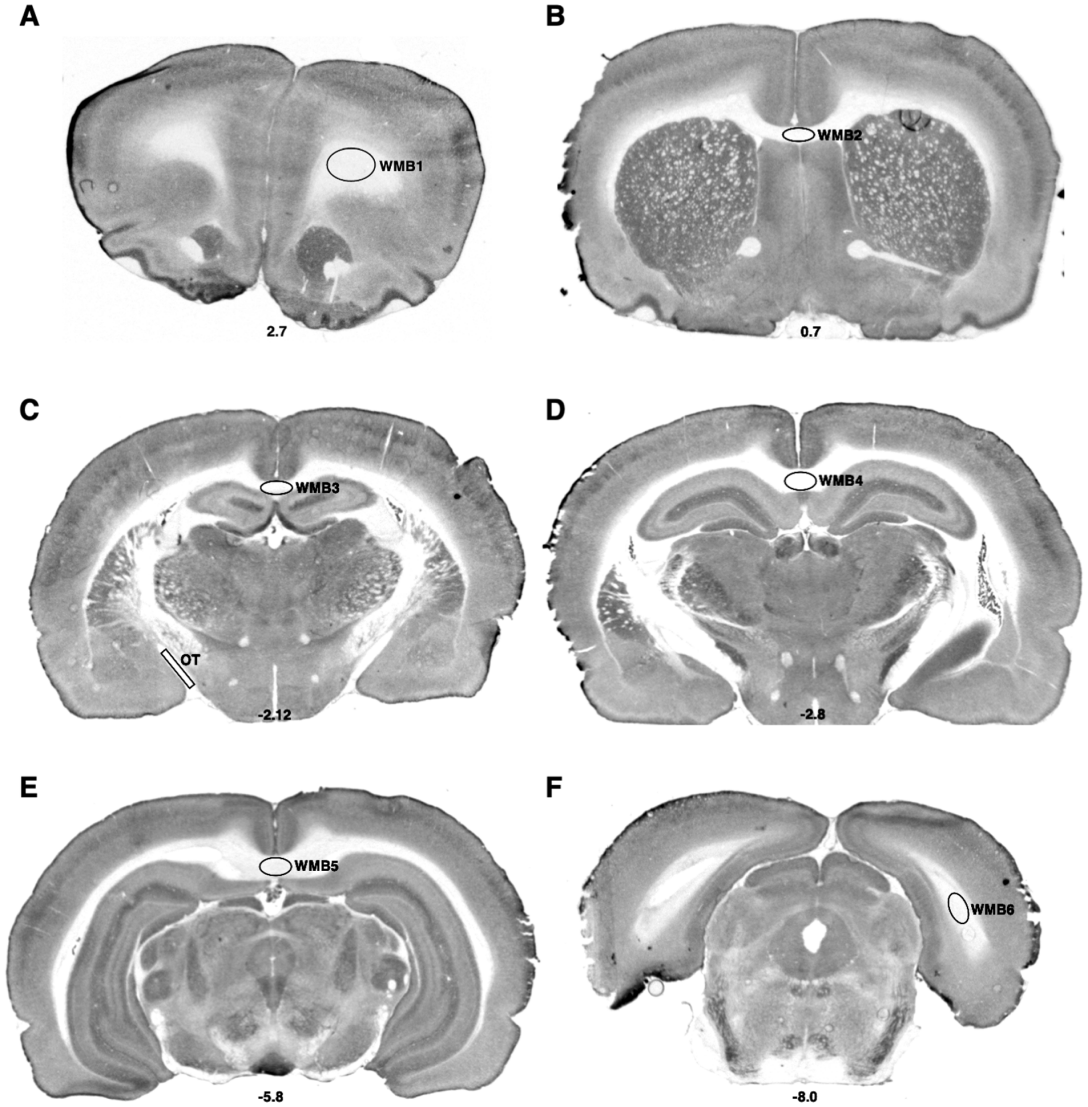
Bregma levels for CCO readings of white matter regions. The Bregma level for each image is shown at the bottom center of that image. Representative images for each Bregma level are shown. **(A)** White matter bundle 1 (WMB1). **(B)** White matter bundle 2 (WMB2). **(C)** White matter bundle 3 (WMB3); optic tract (OT). **(D)** White matter bundle 4 (WMB4). **(E)** White matter bundle 5 (WMB5). **(F)** White matter bundle 6 (WMB6).

Microscope slides of the stained tissue were placed on a light box and the image was captured with a CCD microscope digital camera (Leica Microsystems DFC450, Wetzlar, Germany). To correct for background and optical distortions within the camera, a glass slide with a coverslip adhered with Permount and an optical density (OD) tablet were used to correct for background and create a logarithmic calibration curve relating pixels to OD, respectively. Thirty-nine areas were imaged encompassing sensorimotor (Figure 1), cognitive, and emotional regions (Figure 2), and seven white matter tracks including the corpus collosum (Figure 3) at Bregma levels throughout the brain and the optic track (OT) at Bregma −2.12. Individuals blinded to treatment used ImageJ to quantify the regions of interest by outlining the entire region using a rat brain atlas (Paxinos and Watson, 2006). When possible, OD readings were taken of each region of interest in both hemispheres per subject and 3 brain sections per subject were examined for each region of interest, yielding 5 to 6 readings per subject per region. The readings from these sections were then averaged for each region for an individual animal. In addition, readings from the nucleus accumbens core and shell (Figure 2B) as well as the dentate gyrus at Bregma levels −2.12 and −2.8 (Figures 2C,D) were averaged within each subject. OD values were converted to CCO activity using brain homogenate with known CCO activity determined spectrophotometrically as in O'Reilly et al. (2009). CCO activity is expressed as μmol/min/g wet tissue weight for all regions of interest with the exception of the white matter regions. In these areas, because CCO activity was lower than the range of our standards, OD was averaged across the 5–6 readings for each of the 7 white matter bundles within subject, then reported as 1/OD, or white matter intensity (WMi).

### 2.3 Statistical analysis: regional mean CCO activity

T-tests were performed using RStudio to determine if n-3 PUFA intake affected mean regional CCO activity (T-tests in R, Posit, 2023). All data were normally distributed as determined via Shapiro–Wilk tests. Student’s or Welch’s t-tests were used as appropriate for regions with equal or unequal variances between groups, respectively. Differences between dietary groups were considered significant at *p* < 0.005 as determined using Hochberg’s sharper Bonferroni correction for multiple comparisons (Hochberg, 1988). Cohen’s d was calculated in RStudio as a measure of effect size. Data are expressed as mean ± standard error of the mean μmol/min/g wet tissue weight or, in the case of WMi, 1/OD.

### 2.4 Statistical analysis: structural equation modeling of inter-regional interactions

**Figure 4:**
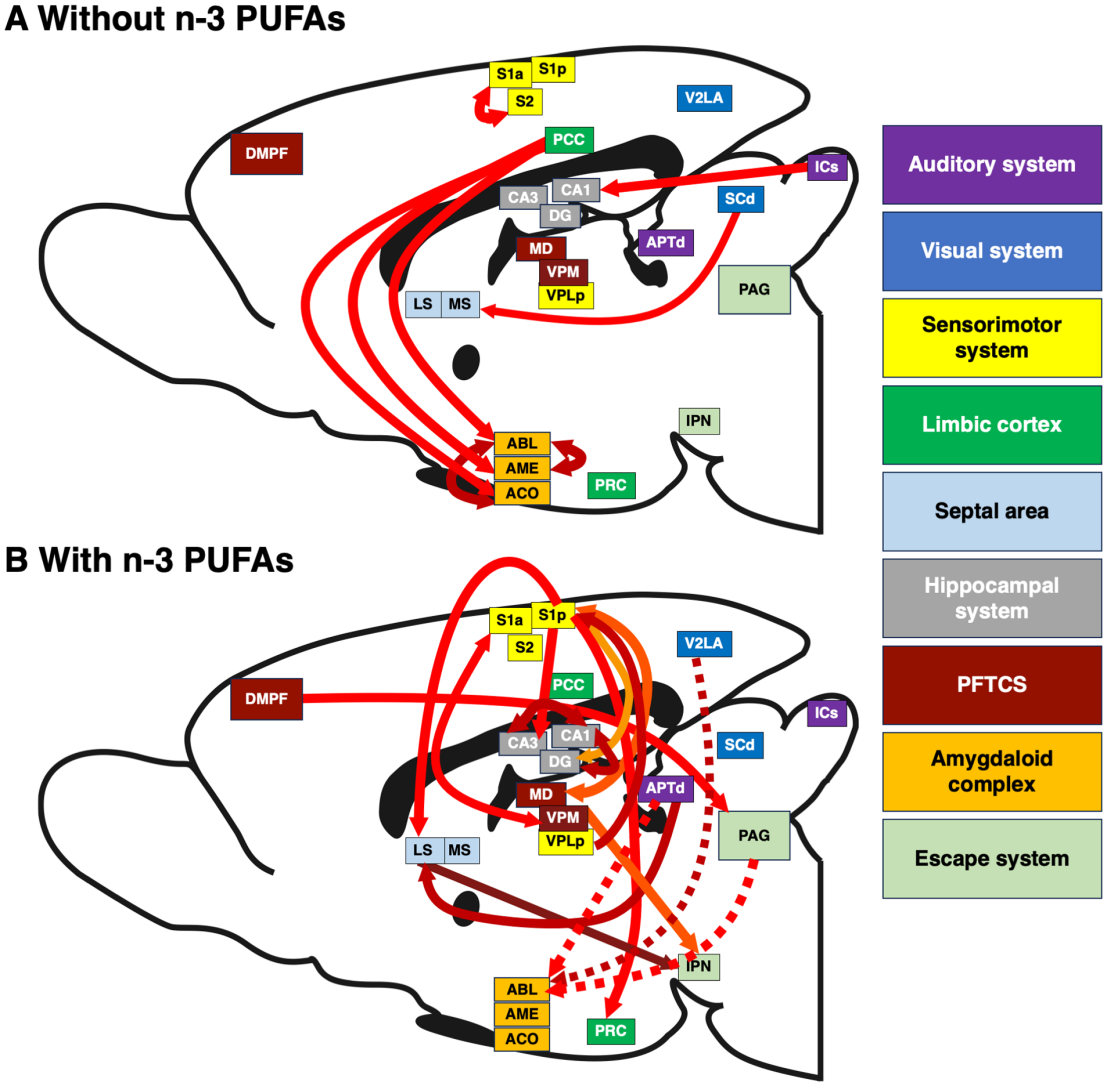
Interactions between brain regions affected by dietary n-3 PUFA consumption. Inter-regional correlations in CCO activity in brains of rats consuming diets **(A)** without and **(B)** with n-3 PUFAs. The width of each arrow is proportional to the inter-regional correlation coefficient. Solid arrows represent positive coefficients, dashed arrows negative. The direction of arrows reflects the predominant path of neurotransmission between pairs of brain regions. Arrow color varies to improve visibility. Because this is a sagittal section in which all relevant regions are flattened to one plane, some cortical regions such as the PRC appear in the center of this image. Sample size is as follows: without n-3 PUFAs, n = 10; with n-3 PUFAs, n = 9.

Correlations in CCO activity between pairs of brain regions, indicative of inter-regional interactions (Sakata et al., 2000; Friston, 2011), were determined using Pearson’s product moment correlations (Supplementary Tables S1, S2). Pairs of regions with strong correlations, indicated by a r with an absolute magnitude ≥0.7 and a Fisher Z-test uncorrected *p*-value <0.01 between dietary groups (Supplementary Table S3, Figure 4) were selected for SEM as in our publication (O'Reilly et al., 2009) and those of our coauthors (McIntosh and Gonzalez-Lima, 1991, 1994; Puga et al., 2007). To eliminate the problem of multiple comparisons, the statistical significance of the interaction between the regions was determined simultaneously using SEM, not the individual pairwise correlations. SEM is a form of causal modeling that in our neural structural models reflected the directionality of known anatomical connections between the regions selected. Modeling was performed using Jamovi (2022). Chi-square tests determined model fit. In each case, the user-defined model fit exhibited a *p* > 0.05, indicating our models fit the data. Some regions in Figure 4 were not included because they were not part of the best fitting model determined by SEM. The baseline models each displayed *p* < 0.05, indicating our user-defined models differed from the null hypothesis model. The comparative fit index (CFI) was used as a measure of how well the user-defined models fit the data. In the functional models, the causal relationships between regions are represented by *β*-coefficients and their corresponding *p*-values, representing the influence the first region has on the second and the significance of that interaction.

### 2.5 Statistical analysis: brain-behavior interactions

To correlate metabolic activity with behavioral outcomes, we used measures of adaptive behavioral coping in the SBET and hyperreactivity to novelty in the open field test (OFT), behaviors affected by n-3 PUFA intake in these animals described in our previous publication (Gonzales et al., 2015). Reduced coping and increased novelty reactivity predict risk for depression-related behavior (Schneiderman et al., 2005; Padilla et al., 2009, 2010; Gonzales et al., 2015; Patel et al., 2019). In the SBET, escape number indicated the number of times each subject successfully completed the trial, terminating footshock within the time limit. Fixed-ratio (FR) 1 trials required a single crossing from one side of the chamber to the other to terminate footshock and lasted up to 15 s. FR2 trials required the rat to cross between sides of the chamber twice to terminate footshock within 30 s. Rats experienced 15 FR1 and 15 FR2 trials. Escape latency was defined as the amount of time it took for the rat to terminate the footshock in the SBET. Higher escape numbers and lower escape latencies indicate improved adaptive coping. In the same study, the novelty reactivity index (NRI) reflects the rat’s exploratory activity in a novel environment on day 1 of the OFT vs. its habituation to the now-familiar environment on day 2 of the OFT, with lower indices indicating reduced hyperreactivity. RStudio was used to perform Pearson’s correlation analyses between escape number and mean FR1 and FR2 escape latency in the SBET as well as the NRI in the OFT and regional CCO activity and WMi. Interactions of interest exhibited a r with an absolute magnitude of ≥0.7. Six region-behavior pairs met this criterion and were subjected to a Fisher Z-test to determine differences due to diet. The Hochberg’s sharper Bonferroni correction (Hochberg, 1988) was applied to correct for multiple comparisons. Differences between groups were considered significant at an uncorrected *p* < 0.006.

## 3 Results

### 3.1 Regional mean CCO activity

N-3 PUFA intake did not affect CCO activity when all gray matter regions were considered together (Table 1). In contrast, n-3 PUFA supplementation resulted in a large increase in WMi (Cohen’s d = 2.710, *p* < 0.0001). WMi is reported as OD^−1^, thus the increase in WMi actually represents a decrease in CCO activity. N-3 PUFA supplementation also resulted in a large decrease in CCO activity in seven sensorimotor regions (Table 2). Two subdivisions of the inferior colliculus (IC) showed decreases: namely, the IC, deep (ICd; Cohen’s d = 2.932, *p* < 0.0001) and IC, superficial (ICs; Cohen’s d = 1.868, *p* = 0.001), suggesting a large negative effect of n-3 PUFA supplementation on CCO activity in the IC. CCO activity was also decreased in two different segments of the primary somatic sensorimotor cortex (S1). Specifically, CCO activity in the S1, anterior measurement (S1a) exhibited a large decrease due to n-3 PUFA supplementation (Cohen’s d = 2.251, *p* < 0.0001), while the S1, anterior barrel field (S1BFa) subregion showed a decrease in CCO activity with a slightly smaller effect size (Cohen’s d = 1.816, *p* = 0.001). Mean CCO activity in the S1, posterior measurement (S1p) was not affected; however, activity in the secondary somatic sensorimotor cortex (S2) was decreased by n-3 PUFA ingestion (Cohen’s d = 1.652, *p* = 0.003). CCO activity in both the secondary visual cortex, lateral, anterior measurement (V2La; Cohen’s d = 1.806, *p* = 0.002) and the ventral posterior nucleus of the thalamus, lateral, anterior measurement (VPLa; Cohen’s d = 1.775, *p* = 0.002) was also reduced in response to consumption of diets with n-3 PUFAs. CCO activity was also lowered by n-3 PUFA intake in one emotional region, the medial amygdala (AME; Cohen’s d = 1.547, *p* = 0.004). Differences in mean CCO activity due to n-3 PUFA intake did not reach significance in any other regions quantified at this conservative, Hochberg’s sharper Bonferroni-corrected *p*-value (uncorrected *p* < 0.005). Together, these data suggest that n-3 PUFA supplementation decreased CCO activity in white matter bundles, several sensorimotor regions, and one region related to emotional processing.

**Table 1:** Effect of n-3 PUFA consumption on brain CCO activity and white matter intensity.

	Bregma level	Without n-3 PUFAs	*n*	With n-3 PUFA	*n*	*p*
All brain regions, white matter excluded^a^	2.70 to −5.80	181.1 [173.1, 189.2]	10	170.7 [162.8, 178.7]	9	0.052
White matter intensity (WMi)^b^	2.70 to −5.80	24.9 [23.7, 26.1]	10	29.7 [28.3, 31.0]	9	<0.000*

a Units represent CCO activity (μmol/min/g wet tissue weight). Data shown are the mean with 95% confidence intervals.

b White matter intensity (WMi) is reported in units of 1/optical density (OD) and averaged across the seven regions measured within each subject.

^*^Significant using Hochberg’s sharper Bonferroni correction at uncorrected *p* < 0.005.

**Table 2:** Effect of n-3 PUFA consumption on regional brain CCO activity, ranked by *p*-value.

	Bregma level	Without n-3 PUFAs	*n*	With n-3 PUFA	*n*	*p*
Sensorimotor regions						
Inferior colliculus, deep (ICd)^a^	−8.0	136.6 [128.1, 145.1]	10	104.7 [97.4, 112.0]	9	<0.000*
Primary somatic sensorimotor cortex, anterior measurement (S1a)	−1.8	178.7 [169.3, 188.2]	10	152.2 [144.7, 159.8]	9	<0.000*
Primary somatic sensorimotor cortex, barrel field, anterior measurement (S1BFa)	−1.8	177.1 [164.5, 189.8]	10	150.8 [143.2, 158.4]	9	0.001*
Inferior colliculus, superficial (ICs)	−8.0	111.1 [96.7, 125.5]	10	74.6 [58.9, 90.3]	8	0.001*
Secondary visual cortex, lateral, anterior measurement (V2La)	−5.8	181.1 [174.5, 187.6]	10	160.9 [149.7, 172.1]	8	0.002*
Ventral posterior nucleus, lateral, anterior measurement (VPLa)	−2.12	169.9 [158.5, 181.2]	9	149.4 [144.1, 154.7]	9	0.002*
Secondary somatic sensorimotor cortex (S2)	−1.8	202.0 [188.9, 215.0]	9	180.5 [175.1, 185.9]	9	0.003*
Primary visual cortex, binocular (V1b)	−8.0	200.5 [188.4, 212.7]	10	179.3 [173.0, 185.6]	8	0.005
Anterior pretectal nucleus, dorsal (APTd)	−5.8	175.3 [167.9, 182.6]	9	160.9 [152.1, 169.7]	9	0.011
Anterior pretectal nucleus, ventral (APTv)	−5.8	141.3 [134.5, 148.1]	9	126.0 [114.4, 137.6]	9	0.018
Ventral posterior nucleus, lateral, posterior measurement (VPLp)	−2.8	122.0 [110.2, 133.9]	10	105.0 [93.6, 116.4]	9	0.031
Superior colliculus, deep (SCd)	−8.0	153.4 [142.1, 164.8]	10	140.0 [133.9, 146.2]	9	0.036
Ventral posterior nucleus, medial (VPM)	−2.8	159.6 [146.0, 173.3]	10	143.2 [128.2, 158.2]	9	0.081
Primary somatic sensorimotor cortex, posterior measurement (S1p)	−2.8	158.4 [146.3, 170.4]	9	147.9 [136.2, 159.7]	9	0.171
Dorsal striatum (STR)	0.7	188.1 [176.1, 200.1]	10	191.9 [176.5, 207.4]	9	0.656
Cognitive and emotional regions						
Medial nucleus of the amygdala (AME)	−2.12	198.0 [184.9, 211.1]	10	175.1 [167.9, 182.3]	9	0.004*
Median raphe nucleus (MRN)	−8.0	166.6 [156.7, 176.5]	10	147.7 [138.3, 157.2]	9	0.006
Posterior cingulate cortex (PCC)	−2.12	245.1 [230.8, 259.3]	10	221.3 [211.9, 230.8]	9	0.007
Dorsal raphe nucleus (DRN)	−8.0	191.1 [173.8, 208.5]	10	167.7 [156.5, 178.9]	9	0.022
Lateral habenula (LH)	−2.8	205.5 [189.3, 221.7]	10	177.5 [154.4, 200.7]	9	0.034
Medial habenula (MH)	−2.8	184.2 [165.2, 203.2]	10	159.7 [144.6, 174.9]	9	0.037
Periaqueductal gray (PAG)	−5.8	174.8 [160.1, 189.5]	10	154.0 [138.6, 169.3]	9	0.039
Basolateral nucleus of the amygdala (ABL)	−2.12	214.1 [197.5, 230.6]	10	195.8 [188.2, 203.4]	9	0.042
Dentate gyrus (DG)	−2.80 and −2.2^b^	228.3 [215.6, 241.1]	10	212.6 [201.5, 223.7]	9	0.051
Mediodorsal nucleus of the thalamus (MD)	−2.8	149.6 [134.5, 164.7]	10	133.9 [123.9, 144.0]	9	0.072
Central nucleus of the amygdala (ACE)	−2.12	202.9 [186.0, 219.8]	10	188.2 [179.8, 196.5]	9	0.106
Medial septal nucleus (MS)	0.7	157.2 [142.6, 171.9]	10	146.7 [136.3, 157.1]	9	0.210
Dorsomedial prefrontal cortex (DMPF)	2.7	165.5 [151.5, 179.5]	10	155.2 [141.1, 169.4]	8	0.258
Cortical nucleus of the amygdala (ACO)	−2.12	193.4 [173.5, 213.3]	10	181.0 [170.5, 191.6]	9	0.244
Hippocampus, CA1 (CA1)	−2.8	110.0 [102.8, 117.3]	10	103.7 [90.3, 117.2]	9	0.346
Prelimbic frontal cortex (PrL)	2.7	157.6 [147.2, 168.0]	10	151.9 [138.8, 165.0]	8	0.437
Rostral linear raphe nucleus (RLi)	−5.8	161.3 [138.3, 184.4]	10	150.4 [131.1, 169.7]	9	0.427
Interpeduncular nucleus (IPN)	−5.8	266.4 [251.7, 281.1]	10	261.2 [241.9, 280.5]	9	0.628
Posterior parietal cortex (PPC)	−2.8	146.6 [137.2, 155.9]	10	144.3 [132.3, 156.3]	9	0.735
Ventral tegmental area (VTA)	−5.8	79.4 [65.6, 93.2]	10	75.3 [45.9, 104.6]	9	0.774
Nucleus accumbens (Acb)	0.7	196.3 [177.2, 215.3]	10	194.1 [176.8, 211.4]	9	0.849
Hippocampus, CA3 (CA3)	−2.8	111.1 [100.1, 122.1]	10	111.6 [97.3, 125.9]	9	0.948
Perirhinal cortex (PRC)	−2.8	132.7 [122.2, 143.3]	10	134.3 [121.2, 147.3]	9	0.837
Lateral septal nucleus (LS)	0.7	206.8 [187.0, 226.7]	10	220.8 [204.5, 237.2]	9	0.237

a Units represent CCO activity (μmol/min/g wet tissue weight). Data shown are the mean with 95% confidence intervals.

b Dentate gyrus CCO activity was averaged at both Bregma levels within each subject.

^*^Significant using Hochberg’s sharper Bonferroni correction at uncorrected *p* < 0.005.

### 3.2 Structural equation modeling of inter-regional interactions

Structural equation modeling (SEM) of neural systems was used to statistically evaluate the network interactions and causal path influences between regions of interest (ROIs) in each of the two treatment groups. SEM is a well-established method for network analysis of metabolic data in neuroscience, including histochemical CCO data of the rat brain, and it involves the construction of a structural model and a functional model (McIntosh and Gonzalez-Lima, 1991, 1994; Puga et al., 2007). The identification of ROIs for the structural models was guided by Figure 4 and Supplementary Table S3, identifying hubs with evidence of strong pairwise inter-regional correlations above an absolute magnitude of r ≥ 0.7 and a Fisher Z-test for effect of diet *p* < 0.01. The final regions shown in the model were determined by the best data fitting model computed by the SEM analysis. The directionality of the interactions in the structural models (causal influence) represents the signal direction of known major anatomical axon connections between these identified brain regions. The functional models used only pairwise correlations of CCO data among these identified ROIs that, after Hochberg’s sharper Bonferroni correction, were found to be still reliably significant at *p* < 0.05. In SEM, a chi-square test is used to statistically compare the user-defined model with a null-hypothesis model in which the covariances of the endogenous variables are set to zero. In each case, the best-fitting models for the two groups were significantly different from the null-hypothesis model at *p* < 0.001.

**Figure 5:**
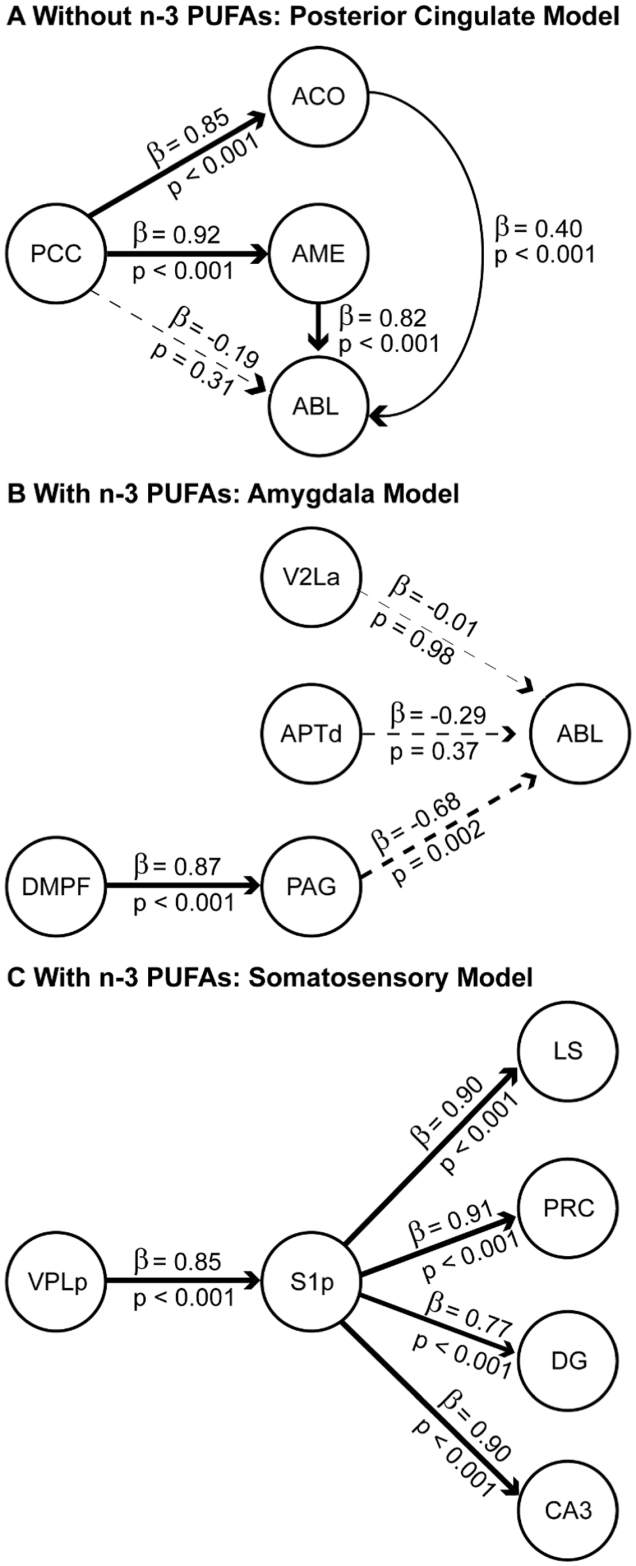
Structural equation models of neural systems impacted by dietary n-3 PUFAs. Regions of interest (ROIs) for the modeling were first identified via pairwise correlations of CCO activity with r of at least 0.7. The resulting optimal data-fitted models are shown for rats consuming diets without **(A)** and with **(B,C)** n-3 PUFAs. Solid lines indicate paths with positive *β*-coefficients and dashed lines indicate paths with negative β-coefficients. A positive path coefficient implies that for each unit of increase in CCO activity in a ROI there is a corresponding unit of increase in the connected ROI. A negative path coefficient implies that for each unit of decrease in CCO activity in a ROI there is a corresponding unit of decrease in the connected ROI. β-Coefficients values are indicated above each line and *p*-values showing statistical significance of the interaction are shown below. The thickness of each line is proportional to the β-coefficient value. Sample size is as follows: without *n*-3 PUFAs, *n* = 10; with n-3 PUFAs, *n* = 9.

The structural model for animals that consumed the diet without n-3 PUFAs consisted of the PCC hub with its direct anatomical connections to ACO, AME, and ABL, and two connections within the amygdala, from ACO and AME to ABL. Based on this network structure, we refer to this model as the “posterior cingulate model.” The functional model (Figure 5A), had a comparative fit index (CFI) =1.00 and simultaneously provided estimates of *β* coefficients that were statistically significant (*p* < 0.001) for four of the five paths in the model: PCC to ACO, PCC to AME, ACO to ABL, and AME to ABL. The greatest influence suggested by the significant β coefficients was from the PCC to the AME.

Networks are represented by two best-fitting structural models for animals ingesting n-3 PUFAs. The first structural model (Figure 5B) used the ABL as a hub, with inputs from V2La, APTd, and PAG, and an additional input from the DMPF to the PAG. Based on this network structure, we refer to this model as the “amygdala model.” The CFI for this model was 0.880. The functional model (Figure 5B) simultaneously provided estimates of β coefficients that were statistically significant (*p* < 0.001) for two of the four paths in the model: DMPF to PAG and PAG to ABL. The greatest influence suggested by the β coefficients was the top-down influence from DMPF to the ABL through the PAG.

The second structural model for animals assigned to the diet with n-3 PUFAs used the S1p as a hub, with outputs to the LS, PRC, DG, and CA3 and an input from VPLp to S1p. Based on this network structure, we refer to this model as the “somatosensory model.” The CFI for this model was 0.966. The functional model (Figure 5C) simultaneously provided estimates of β coefficients that were statistically significant (*p* < 0.001) for all five paths in the model. The model suggested large influences of somatosensory cortex (S1p) on the identified limbic regions.

### 3.3 Brain-behavior interactions

**Figure 6:**
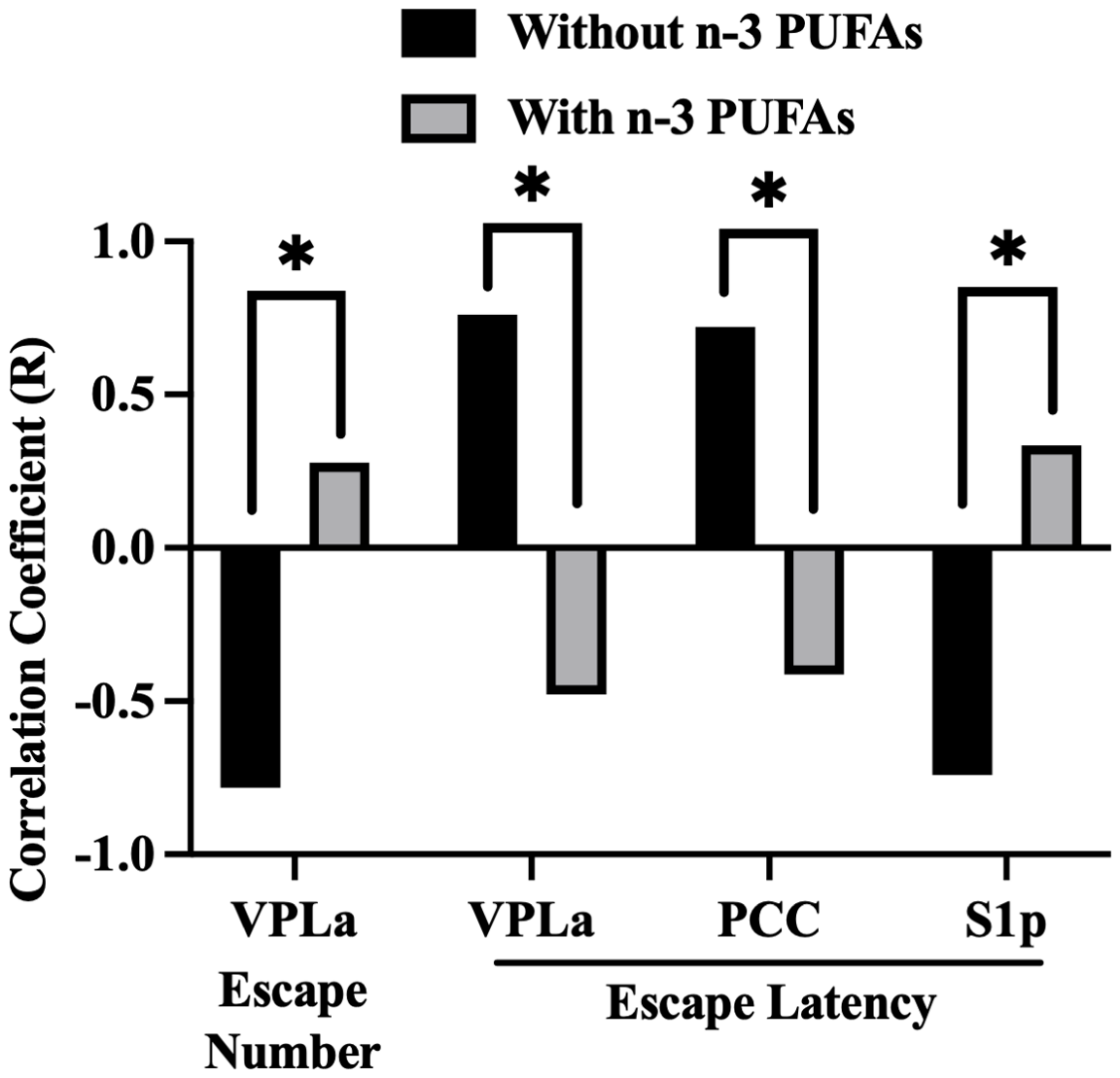
Impact of dietary n-3 PUFAs on interactions between behavior and regional CCO activity. Escape number refers to the total number of escapes out of 30 SBET trials. FR2 escapes required the animal to cross between chambers twice to terminate footshock. The FR2 escape latency was averaged across 15 FR2 trials. Comparison of Pearson’s correlation coefficients between n-3 PUFA deficient and supplemented groups, n = 10 without and n = 9 with n-3 PUFA. *Significant using Hochberg’s sharper Bonferroni correction at uncorrected *p* < 0.006.

In our previous study (Gonzales et al., 2015), we showed that n-3 PUFA supplementation resulted in increased escape number and decreased escape latency in the SBET as well as reduced NRI in the OFT. To determine if these behaviors were associated with altered CCO activity, we examined correlations between SBET escape number, average FR1 and FR2 escape latency, the NRI, and regional CCO activity, whole brain CCO activity, and WMi. Six behavior-region pairs exhibited r with an absolute magnitude ≥0.7. After correction for multiple comparisons, Fisher Z-tests revealed that diet altered the interaction between behavior and regional CCO activity in 4 cases (Figure 6). Higher escape number reflects improved performance in the SBET, while increased escape latency indicates poorer SBET performance. SBET escape number was negatively correlated with CCO activity in the VPLa in animals that consumed diets without n-3 PUFAs. This correlation was not observed in animals that consumed diets with n-3 PUFAs. VPLa CCO activity was also positively associated with FR2 escape latency in animals ingesting n-3 PUFAs, but no relationship was observed in those lacking dietary n-3 PUFAs. A similar relationship was observed between CCO activity in the PCC, FR2 escape latency, and dietary n-3 PUFAs. CCO activity in the S1p exhibited a negative relationship with FR2 escape latency in animals consuming diets with n-3 PUFAs that was not observed in the absence of n-3 PUFAs. Taken together, in the absence of dietary n-3 PUFAs, higher CCO activity in the VPLa and PCC are associated with reduced performance in the SBET. In contrast, reduced S1p CCO activity is associated with lower escape latency indicating improved performance in this test.

## 4 Discussion

**Figure 7:**
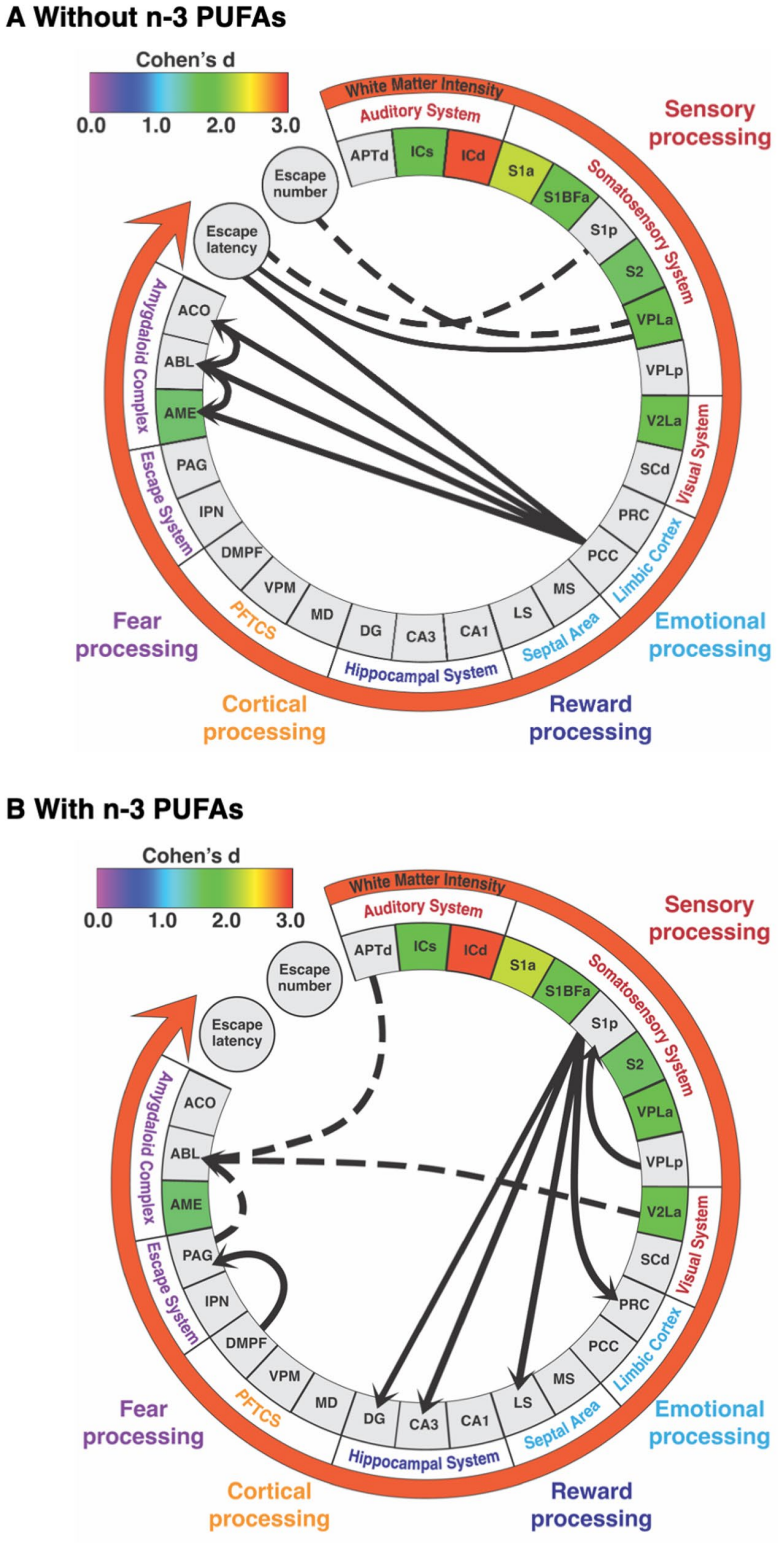
Summary of alterations in brain white matter and regional metabolic capacity, inter-regional interactions, and brain-behavior correlations in response to diets **(A)** without and **(B)** with n-3 PUFAs. Regions are grouped by functional system. Regions included are those in which inter-regional correlations were likely to be affected by diet. Colored boxes indicate regions in which mean CCO activity was decreased when rats consumed diets with n-3 PUFAs. The effect size (Cohen’s d) is indicated by the color of these boxes and the white matter intensity arrow as per the calibration bar. Solid lines connecting regions, escape latency, and escape number represent positive correlation coefficients while dashed lines indicate negative coefficients. Lines between regions reflect functional path interactions within each dietary group that were assessed by structural equation modeling. Abbreviations are as in Table 2 except for prefrontal thalamocortical system (PFTCS).

Previously, we showed that dietary n-3 PUFA supplementation increased behavioral coping, or the ability to learn under stress, in postpartum rats (Gonzales et al., 2015). The current study systematically examined how dietary n-3 PUFAs affected regional brain energy metabolic capacity using CCO histochemistry. Unique aspects of the current study include the use of adult, female animals, a systemic examination of white matter tracks as well as 39 brain regions spanning multiple functional systems, and the ability to link oxidative energy metabolism to behavioral outcomes. Figure 7 summarizes our results and illustrates the pervasive effects of this dietary intervention. We found that ingestion of n-3 PUFAs increased WMi, corresponding to reduced CCO activity. In addition, dietary n-3 PUFA supplementation reduced CCO activity in several sensorimotor regions, specifically the deep and superficial IC, the S1a, the S1BFa, the V2La, and the VPLa as well as one limbic region, the AME. Furthermore, brains from rats consuming diets with n-3 PUFAs exhibited different network interactions, elucidated via SEM, than rats lacking these essential fatty acids in their diet. We also identified brain-behavior correlations between CCO activity in the VPLa, PCC, and S1p and escape behavior in the SBET in animals consuming diets without n-3 PUFAs but not in those with n-3 PUFAs. The absence of correlations between regional CCO activity and behavior in the “with” group supports our hypothesis that consumption of n-3 PUFAs resulted in more functionally-efficient brain systems, perhaps due to more efficient signal conduction through white matter, resulting in a more synchronous pattern of neuronal activity, facilitating the improved spatial and sensory awareness associated with successful coping behavior.

### 4.1 N-3 PUFAs decrease CCO activity in white matter bundles, reflecting increased efficiency

N-3 PUFA supplementation increased WMi, indicating a decrease in CCO activity. The white matter CCO activity associated with n-3 PUFA supplementation reflects increased energy efficiency likely due to improved signal conduction through myelinated white matter. This novel finding helps to explain how n-3 PUFAs effect behavior because more efficient signal transduction facilitates communication between neurons. Reduced CCO activity reflects increased efficiency and decreased energy demand (Wong-Riley, 2012). As axons become more myelinated, there is less surface area for ion exchange. Transmembrane ion exchange consumes the majority of ATP generated via the electron transport chain coupled with oxidative phosphorylation. CCO is the rate limiting enzyme in the electron transport chain. The myelin insulates the axon to reduce and isolate ionic exchange to nodes of Ranvier, termed “saltatory conduction.” Saltatory conduction is faster due to this insulation. Because the ion exchange is limited to the nodes of Ranvier, less ATP is needed, as reflected in reduced CCO activity. This phenomenon occurs during postnatal development as axons mature and become myelinated, more energy efficient, and exhibit lower CCO activity (Nair et al., 1999).

There is ample evidence that n-3 PUFAs improve myelination. For example, n-3 PUFAs increase the expression of myelinogenic mRNAs (Salvati et al., 2008), protect cultured oligodendrocytes from excitotoxicity (Pu et al., 2013), reduce axonal dysfunction (Ward et al., 2010), prevent axon injury (Bailes and Mills, 2010), and reduce demyelination (Ward et al., 2010; Chen et al., 2014), indicating that overall these lipids improve white matter integrity (Jiang et al., 2016). In support of this, studies using diffusion tensor imaging show a positive relationship between n-3 PUFA intake and improved microstructural integrity of white matter in healthy elderly individuals (Gu et al., 2016), patients with MDD (Chhetry et al., 2016), and those with recent-onset schizophrenia (Peters et al., 2009). Interestingly, in previous studies assessing CCO activity, we have not observed changes in the activity of this enzyme in white matter bundles in rodents treated with 13-*cis*-retinoic acid, fluoxetine, or methylene blue (O'Reilly et al., 2009; Padilla et al., 2011; Riha et al., 2011), indicating this phenomenon is unique to n-3 PUFAs. The fundamental role of myelin in the central nervous system and the unique ability of n-3 PUFAs to improve myelin may underscore the plethora of neurological benefits attributed to these essential lipids.

### 4.2 N-3 PUFAs decrease CCO activity in somatosensory regions and the medial amygdala

The current study simultaneously and systematically assessed the impact of dietary n-3 PUFA status on multiple brain regions representing a repertoire of functions. This neuroscience systems approach allowed us to elucidate the wide-spread effects of n-3 PUFAs throughout the brain, contrasting prior research focusing on relatively few brain regions per study. We show that n-3 PUFA supplementation decreased CCO activity in seven somatosensory and one emotional region: the inferior colliculus (ICd and ICs), visual cortex (V2La), S1 and S2 cortices (S1a, S1BFa, S2), thalamus (VPLa), and amygdala (AME). As mentioned above, decreased CCO activity reflects decreased neuronal firing and increased regional efficiency. The regions exhibiting reduced CCO activity in response to n-3 PUFA consumption process or are activated by sensory stimuli and contribute to our understanding regarding how n-3 PUFA consumption improved coping behavior in our previous study (Gonzales et al., 2015). Specifically, these regions function together as follows: The IC receives auditory stimuli from both ears and some visual stimuli from the superior colliculus before transmitting these impulses to the medial geniculate nucleus of the thalamus. The thalamus receives input from the periphery, relaying stimuli to the auditory, visual, and somatosensory cortices ultimately activating the amygdala. For example, the quick pain of a footshock, the relevant stimulus in this study, enters the S1 through myelinated afferents suggesting that myelination is crucial for the S1 to receive pain stimuli. The S2 processes incoming stimuli from the S1. The AME is strongly activated by stressful stimuli including auditory stimuli such as ultrasound stress and footshock (reviewed in Zhang et al., 2021).

Earlier work found that dietary fish oil supplementation from conception to adulthood increased CCO subunit VIc mRNA expression in rat whole brain homogenates (Kitajka et al., 2004). One reason for these differences may be due to altered n-3 PUFA status beginning at conception as opposed to in adult animals as in the current work. In work using adult rats, administration of fish oil increased CCO activity in cerebral cortex homogenates (Chomova et al., 2017); however, use of homogenates eliminates the ability to examine regional CCO activity. In support of our findings, others have shown that n-3 PUFA intake affects auditory nerve conduction in rodents (Rahimi et al., 2014) and hearing thresholds in humans, particularly women (Long et al., 2022). In a perinatal ischemic injury model, DHA restored IC morphology and, by increasing myelination, auditory function (Revuelta et al., 2016). With respect to vision, functional connectivity from the retina to the primary visual cortex was lost in macaques with a life-long deficiency in n-3 PUFAs (Grayson et al., 2014). N-3 PUFAs also prevented an increase in inflammatory markers in the amygdala of mice exposed to cold stress (Lin et al., 2020). It is unusual to have multiple significant t-test *p*-values with CCO histochemistry, particularly with the strict criteria used here to account for multiple comparisons (Shumake and Gonzalez-Lima, 2003; O'Reilly et al., 2009; Rojas et al., 2009; Riha et al., 2011; Vélez-Hernández et al., 2014). This demonstrates the profound and widespread effect of n-3 PUFAs on neural CCO activity, manifested in these rats as a better ability to process sensory stimuli, emotions like fear, and escape the stress of footshock.

### 4.3 N-3 PUFAs increase the number of inter-regional interactions

We used SEM to determine how dietary n-3 PUFAs alter interactions between brain regions. This analysis revealed three models: (1) the “posterior cingulate model” present in deficient animals as well as (2) the “amygdala model” and (3) “somatosensory model,” present in animals consuming n-3 PUFAs. Perhaps the most striking effect of dietary n-3 PUFA status on inter-regional interactions in our study concerned the amygdaloid complex. Specifically, our “posterior cingulate model” showed that brains obtained from rats deficient in n-3 PUFAs exhibited strong, direct causal interactions, between the PCC, ACO, and AME as well as within the amygdala itself, among the ACO and ABL and to a more moderate extent, between the ACO and ABL. Two studies have shown that n-3 PUFAs increase inter-regional interactions between a limited number of brain regions in adult, obese mice (Arnoldussen et al., 2016) and depressed, middle-aged women (Park et al., 2020); however, behavior was not assessed in these studies. In contrast, much is known about how the regions identified in our model, the PCC and amygdala, affect reward processing and the stress response.

The PCC is multifunctional, densely connected, and highly affected by variabilities in white matter (reviewed in Leech and Sharp, 2014). The PCC is involved in fear memory consolidation, spatial processing, as well as pain perception and processing (Bastuji et al., 2016; Garcia-Larrea and Bastuji, 2018). Interestingly, activation of the PCC leads to errors in reward prediction (Dickhaut et al., 2003). Escape from footshock, assessed in the SBET in our prior publication (Gonzales et al., 2015), can be viewed as a reward. Indeed, this is reflected in the positive relationship between CCO activity in the PCC and SBET escape latency in n-3 PUFA deficient animals, discussed below. In humans, fMRI results indicating altered inter-regional interactions between the PCC and amygdala are associated with post-partum depression and anxiety (Chase et al., 2014; Toazza et al., 2016). This is relevant because, although dietary n-3 PUFAs did not impact behavioral despair in these rats (Gonzales et al., 2015), our study design reflects other studies attempting to model the role of n-3 PUFAs on postpartum depression in rats (Levant et al., 2006, 2007, 2008). Rats in the without n-3 PUFA group did display a reduction in coping, the adaptive behavioral response to stress (Gonzales et al., 2015), reflective of the role of the amygdala in the stress response. For example, hyperexcitability of the amygdala predicts stress behaviors in response to stressors (reviewed in Zhang et al., 2018). Also, in older adult humans, higher resting amygdala cerebral blood flow was associated with reduced resilience to stress, particularly in the ABL of individuals with MDD (Rabinak et al., 2011; Leaver et al., 2018). These studies support our work showing that when rats consume diets without n-3 PUFAs, the inter-regional interactions between the PCC and amygdala as well as the increased interactions within the amygdala itself contributed to the reduced escape behavior from footshock, or failed reward, observed in our prior study (Gonzales et al., 2015).

Our “amygdala model” assessed causal inter-regional influences between the V2La, APTd, PAG, and ABL in rats consuming diets with n-3 PUFAs. In addition, the influence of the DMPF, representing the prefrontal cortex, on the PAG was also determined. The V2La processes visual stimuli (Mertens et al., 2023). Like the V2La, the APTd is involved in vision but this region is also antinociceptive, inhibiting pain (reviewed in Murray et al., 2010; Genaro and Prado, 2021). The PAG is also associated with pain perception (reviewed in Mohammadi et al., 2022) and is active during reactive escape (Jing et al., 2023), as in the SBET. The ABL both acquires and regulates the response to conditioned fear (Gale et al., 2004), expressed as freezing. Finally, the DMPF is implicated in spatial memory (Kitamura et al., 2017). We found that the V2La had almost no causal influence on the ABL, the APTd exerted a modest, inhibitory influence on the ABL, and the primary, inhibitory direct causal influence on the ABL was exerted by the PAG. We also found that the DMPF had an inhibitory influence on the ABL, mediated through the PAG. To our knowledge, little work exists regarding the impact of n-3 PUFAs on these regions and escape or reward behavior. One study showed that dietary n-3 PUFAs increase cannabinoid receptor sensitivity in the ABL, resulting in decreased freezing in response to a conditioned stimulus (Yamada et al., 2014). Others have indicated that n-3 PUFAs reduce pain in humans (Abdulrazaq et al., 2017; Mohammadi et al., 2022; Deng et al., 2023) and mice (Liao et al., 2024). Taken together, these results may indicate that the causal influences between the DMPF and PAG as well as the negative relationships between PAG and APTd with the ABL seen in the rats consuming diets with n-3 PUFAs reflects a focusing of attention away from the sensory stimuli, like pain, that evoke emotion, resulting in less fear and reduced freezing. As a result, these animals were better able to perceive the escape route in the SBET, a behavior associated with active coping.

Active coping strategies develop because, during a stressful event, a short-term memory of the experience is created due to the strength of synapses firing between the hippocampus and the neocortex, particularly in areas that register the stressor (Kitamura et al., 2017). Our “somatosensory model” reflects this, showing strong, direct causal inputs from the thalamus (VPLp) to the S1p and from the S1p to the LS, PRC, as well as the hippocampal DG and CA3 regions in rats consuming n-3 PUFAs. This suggests that the thalamus (VPLp) via the S1p exerted a causal influence on CCO activity in the LS, PRC, and hippocampus. With the exception of the hippocampus, information is lacking regarding how n-3 PUFAs affect these regions. Interestingly, CCO activity in the LS is associated with increased coping and decreased learned helplessness (Shumake et al., 2002) while the PRC and hippocampus both contribute to item-location associated memory (Yang and Naya, 2023). In the case of the SBET, the item may have been the door connecting chambers, through which the rats must escape to terminate footshock. This “somatosensory model” and the above-mentioned “amygdala model” in the “with n-3 PUFA” group may reflect a more synchronized pattern of brain activity, discussed below, facilitating improved spatial awareness during stress leading to adaptive coping techniques and memory formation.

### 4.4 N-3 PUFAs alter the relationship between regional CCO activity and escape behavior

In animals consuming diets without n-3 PUFAs, the VPLa exhibited higher mean CCO activity than in those consuming diets with n-3 PUFAs. In these animals increased VPLa activity is also correlated with fewer escapes from footshock and increased escape latency, both of which are worse behavioral outcomes in the SBET. This relationship is not observed when n-3 PUFAs are consumed. A similar pattern is observed between the PCC and escape latency. As mentioned above, this may reflect the role of the PCC in reward processing. Of note in the “without” group, increased CCO activity in the S1p was correlated with improved escape behavior. This suggests that increased sensation of footshock, reflected in elevated CCO activity in the S1p, resulted in faster, more frequent escapes. In other words, rodents in the “without” group were using bottom-up processing to respond to, rather than anticipate, the noxious stimulus.

In bottom-up processing, the brain and spinal cord both create and modulate pain perception, while central processing allows for the conscious perception of pain. Acute pain, as in footshock, is transferred from peripheral nociceptors through the dorsal horn of the spinal cord to the brainstem and ventral posterior thalamus, in the current study represented by the VPLa, VPLp, VPM. The information is then transferred to the insula, somatosensory cortices (represented here by the S1a, S1p, S1BFa), cingulate cortices (PCC), the basal ganglia, parietal cortices (PPC), the amygdala (AME, ABL, ACE, ACO), the hypothalamus, and prefrontal cortices (DMPF). Together, these regions are known as the pain matrix. There is also a descending, top-down pathway that controls pain perception. In the PAG, ascending pain stimuli and descending cortical influences, primarily from the hypothalamus, amygdala, rostral anterior cingulate cortex, insula, and orbitofrontal cortex, are integrated to induce analgesia via connection to the rostral medulla. Pain, for example from footshock, is processed in both a bottom-up and top-down fashion (Ruff, 2013).

While the antinociceptive effects of n-3 PUFAs are beginning to be recognized, it is unclear whether this reduction in pain is due to inhibition of bottom up or activation of top-down pain processing. A recent study using a mouse fibromyalgia model showed n-3 PUFAs inhibiting the bottom-up pain pathway, reducing pain (Liao et al., 2024). When considered together, this study, coupled with the correlations between regional CCO activity and escape behavior as well as the SEM results, suggest that rats consuming diets lacking n-3 PUFAs in the current study relied on bottom-up processing due to the relative lack of inter-regional interactions, particularly with the PAG, in contrast to rats consuming n-3 PUFAs, discussed in the preceding section. The use of top-down processing in rats consuming n-3 PUFAs is supported by the increased inter-regional interactions with the S1p in this group potentially demonstrating a shifting of attention towards the S1p in preparation of the next footshock, reflected in the behavior of these animals (Gonzales et al., 2015). Specifically, rodents in the “with” group had an overall increase in escape number and decreased escape latency throughout the SBET. Escape latency decreased with each subsequent trial, perhaps demonstrating that preparatory activity of the S1p increased the speed by which the rodent escaped, reflecting the increased efficiency of the brains of rats consuming diets with n-3 PUFAs.

### 4.5 N-3 PUFA supplementation results in a more global pattern of inter-regional interactions

This study showed that systemic, chronic dietary manipulation results in changes in network interactions, shifting from a model including two regions, the PCC and amygdala, in n-3 deficient animals to models encompassing 10 regions with a variety of functions in supplemented rats. We believe the greater number of inter-regional interactions in animals that consumed n-3 PUFA resulted in a more global (also referred to integrated or synchronized) functional network of inter-regional interactions allowing rats, and by extension humans, to learn adaptive responses to stressful situations. Global and local (i.e., segregated or desynchronized) functional networks facilitate different aspects of cognition. During a task, local or segregated/desynchronized network communication is required for motor skills, while global or integrated/synchronized, between-network communication is required for working memory (Cohen and D'Esposito, 2016). In support of this, restoration of frontotemporal synchronization in elderly patients experiencing cognitive decline improved working memory (Reinhart and Nguyen, 2019). Similar patterns exist at rest. For example, analysis of healthy human subjects revealed that more integrated, synchronized brains are associated with higher general cognition while segregated, desynchronized brains reflect more crystallized intelligence and higher processing speed (Wang et al., 2021). A balance between integration and segregation is believed to be ideal, supporting memory and allowing for efficient switching between tasks. Our finding that n-3 PUFAs produce a more global pattern of inter-regional interactions is supported by other work. Specifically, in non-human primates, n-3 PUFA deficiency from birth through adulthood resulted in a reduction of inter-regional interactions and an abnormal, more localized, whole-brain functional connectivity structure, when compared to DHA supplementation (Grayson et al., 2014). Similarly, in healthy, elderly humans n-3 PUFAs affected a variety of brain regions and increased global functional network connectivity (Talukdar et al., 2019). Although these studies did not assess behavior, the ability of n-3 PUFAs to shift functional networks towards more a global pattern deserves more exploration as it may explain many of the cognitive phenomena supported by n-3 PUFAs.

## 5 Conclusion

In conclusion, our results indicate that dietary supplementation of adult, female rats with n-3 PUFAs resulted in more efficient energy metabolism in white matter, impacted a variety of sensorimotor regions, and one region of the amygdala. N-3 PUFAs also increased inter-regional interactions between brain regions, resulting in a more integrated, synchronized pattern of brain connectivity, perhaps facilitated by more efficient signal conduction through white matter. Taken together these alterations support the improved adaptive escape response to stress observed in rats consuming n-3 PUFAs. Future studies should quantify the impact of dietary n-3 PUFAs on myelination as well as processing and perception of the pain of footshock. This study highlights the beneficial effects of n-3 PUFAs, a nutrient often consumed at suboptimal levels.

## Data Availability

The raw data supporting the conclusions of this article will be made available by the authors, without undue reservation.
