# Supplementary material for: Dietary omega-3 polyunsaturated fatty acids reduce cytochrome c oxidase in brain white matter and sensorimotor regions while increasing functional interactions between neural systems related to escape behavior in postpartum rats
Source: Front Syst Neurosci. 2024 Oct 31;18:1423966. doi: 10.3389/fnsys.2024.1423966 (PMC11560429; doi:10.3389/fnsys.2024.1423966)
Supplement: Supplementary file 3 [file Table_3.DOCX]

**Supplemental Table 3.** Interregional correlations altered by dietary n-3 PUFA intake.

|  |  | ***With n-3 PUFAs*** | | ***Without n-3 PUFAs*** | |  |
| --- | --- | --- | --- | --- | --- | --- |
| **First region** | **Second region** | **Pearson's Correlation Coefficient (r)** | **Pearson's Correlation p-value** | **Pearson's Correlation Coefficient (r)** | **Pearson's Correlation p-value** | **Fisher Z-test uncorrected**  **p-value** |
| *Correlation present in "with" group; correlation absent in "without" group* | | | | | | |
| ABL^1^ | APTd | -0.850 | 0.004 | 0.055 | 0.888 | 0.0054 |
| ABL | V2La | -0.773 | 0.024 | 0.243 | 0.498 | 0.0071 |
| CA1 | CA3 | 0.965 | <0.000 | 0.457 | 0.185 | 0.0009 |
| CA1 | DG | 0.836 | 0.005 | -0.213 | 0.554 | 0.0019 |
| CA3 | S1p | 0.903 | 0.001 | 0.122 | 0.754 | 0.0038 |
| DG | S1p | 0.764 | 0.016 | -0.440 | 0.236 | 0.0017 |
| DMPF | PAG | 0.874 | 0.005 | 0.099 | 0.303 | 0.0084 |
| IPN | VPM | 0.932 | <0.000 | 0.208 | 0.564 | 0.0015 |
| LS | APTd | 0.876 | 0.002 | -0.121 | 0.756 | 0.0017 |
| LS | IPN | 0.826 | 0.006 | -0.092 | 0.801 | 0.0058 |
| LS | S1p | 0.903 | 0.001 | -0.258 | 0.503 | 0.0002 |
| MD | S1p | 0.939 | <0.000 | -0.246 | 0.524 | 0.0000 |
| PAG | ABL | -0.890 | 0.001 | -0.069 | 0.850 | 0.0032 |
| PRC | S1p | 0.912 | 0.001 | 0.065 | 0.867 | 0.0018 |
| S1a | VPM | 0.814 | 0.008 | -0.092 | 0.800 | 0.0074 |
| VPLp | S1p | 0.852 | 0.004 | -0.034 | 0.930 | 0.0059 |
| *Correlation absent in "with" group; correlation present in "without" group* | | | | | | |
| ABL | ACO | -0.004 | 0.993 | 0.905 | <0.000 | 0.0011 |
| ABL | AME | 0.444 | 0.231 | 0.962 | <0.000 | 0.0011 |
| CA1 | ICs | -0.047 | 0.728 | 0.851 | 0.002 | 0.0059 |
| MS | SCd | -0.340 | 0.370 | 0.707 | 0.022 | 0.0072 |
| PCC | ABL | 0.168 | 0.666 | 0.885 | 0.001 | 0.0075 |
| PCC | ACO | 0.002 | 0.997 | 0.851 | 0.002 | 0.0062 |
| PCC | AME | 0.208 | 0.592 | 0.916 | <0.000 | 0.0032 |
| S1a | S2 | 0.202 | 0.602 | 0.935 | <0.000 | 0.0016 |

1. Abbreviations are as in Figure 1 and Table 2; Pearson's correlation coefficients are shown for n = 10 (without n-3 PUFAs) and n = 9 (with n-3 PUFAs).
